# Water Deficit and Methyl Jasmonate Enhance the Antiplatelet Potential of Blueberries Through Changes in Selected Phenolic Compounds

**DOI:** 10.3390/ijms27167306

**Published:** 2026-08-16

**Authors:** Carlos Vasquez-Rojas, Lyanne Rodríguez, Daniel Bustos, Valentina Jara-Villacura, Cristian Balbontín, Gabriela Urra, Ricardo E. Hernández, Evelyn Villagra, Daniel Laporte, Carolina Parra-Palma, Patricio Ramos, Eduardo Fuentes, Luis Morales-Quintana

**Affiliations:** 1 Multidisciplinary Agroindustry Research Laboratory, Instituto de Ciencias Biomédicas, Facultad de Ciencias de la Salud, Universidad Autónoma de Chile, Cinco Poniente #1670, Talca 3460787, Chile; carlos.vasquez6@cloud.uautonoma.cl (C.V.-R.); vjaravillacura@gmail.com (V.J.-V.); carolina.parra@uautonoma.cl (C.P.-P.); 2 Programa de Doctorado en Ciencias Biomédicas, Facultad de Ciencias de la Salud, Universidad Autónoma de Chile, Santiago 8580746, Chile; 3 Thrombosis and Healthy Aging Research Center, VITALIS Longevity Center, Medical Technology School, Faculty of Health Sciences, Interuniversity Center for Healthy Aging, Universidad de Talca, Talca 3460787, Chile; lyrodriguez@utalca.cl; 4 Laboratorio de Bioinformática y Química Computacional, Departamento de Medicina Traslacional, Facultad de Medicina, Universidad Católica del Maule, Talca 3480094, Chile; dbustos@ucm.cl; 5 Instituto de Investigaciones Agropecuarias, INIA Quilamapu, Av. Vicente Méndez 515, Chillán 3800062, Chile; cristian.balbontin@inia.cl; 6 Doctoral Program in Sciences with a Specialization in Modeling of Chemical and Biological Systems, Faculty of Engineering, Universidad de Talca, Talca 3460000, Chile; gabriela.urra@utalca.cl; 7 Laboratory of Phytochemistry, Centre of Biotechnology in Natural Resources, Faculty of Agricultural and Forestry Sciences, Universidad Católica del Maule, Ave. San Miguel, 3605, Talca 3480112, Chile; ricardo.hernandez@alu.ucm.cl (R.E.H.); evillagra@ucm.cl (E.V.); 8 Laboratory of Plant Physiology and Molecular Biology, Instituto de Ciencias Biomédicas, Facultad Ciencias de la Salud, Universidad Autónoma de Chile, Talca 3460787, Chile; daniel.laporte@uautonoma.cl; 9 Plant Microorganism Interaction Laboratory, Instituto de Ciencias Biológicas, Universidad de Talca, Talca 3460787, Chile; pramos@utalca.cl

**Keywords:** antioxidant capacity, drought stress, functional food, nutritional quality

## Abstract

Agronomic modulation of secondary metabolism may influence not only crop resilience but also the biological activity of fruit-derived phytochemicals. In this study, we evaluated the impact of exogenous methyl jasmonate (MeJA) application under contrasting water regimes on the selected phenolic compounds and vascular bioactivity of *Vaccinium corymbosum* L. cv. Legacy. Antioxidant capacity was assessed by FRAP and DPPH assays, phytochemical composition was characterized by HPLC-DAD, and antiplatelet activity was evaluated through inhibition of TRAP-6–induced P-selectin (CD62P) expression in human platelets. Selected phenolic constituents were further examined using molecular docking and molecular dynamics simulations against a platelet receptor model. Although MeJA treatment altered the abundance of selected phenolic compounds identified by HPLC-DAD, total antioxidant capacity remained largely unchanged. Blueberry extracts significantly inhibited platelet activation in a concentration-dependent manner without cytotoxic effects, and antiplatelet potency was not strictly related to global antioxidant indices. Computational analyses revealed stable ligand–receptor interactions and favorable binding free energies for selected phenolics, providing a structural explanation for receptor-level modulation. These findings suggest that elicitor-driven responses in blueberries can influence platelet functional responses and highlight the importance of qualitative phytochemical composition in determining vascular bioactivity. This multiscale approach connects plant stress physiology, natural product chemistry, and human platelet biology, underscoring the translational relevance of agronomic strategies for nutraceutical functionality.

## 1. Introduction

Thrombo-inflammatory and cardiovascular pathologies are predominantly mediated by oxidative stress, a pathophysiological condition characterized by excessive generation of reactive oxygen species (ROS) that perturbs redox-sensitive signal transduction cascades governing endothelial homeostasis, inflammatory responses, and hemostatic processes [1]. Sustained redox imbalance promotes post-translational modifications of receptor proteins, disrupts intracellular signaling networks, and alters protein–ligand binding affinities, collectively facilitating aberrant platelet activation and pathological thrombus formation [2]. In this context, platelet activation is orchestrated through precisely regulated receptor-mediated signaling pathways, among which the adenosine A_2_A receptor (ADORA2A), a class A G protein-coupled receptor (GPCR), plays a central inhibitory role. Upon activation, ADORA2A couples to Gαs proteins, stimulating adenylyl cyclase activity, increasing intracellular cyclic adenosine monophosphate (cAMP) levels, and consequently attenuating platelet aggregation, granule secretion, and cytoskeletal reorganization [3,4]. Disruption of this inhibitory axis under oxidative and inflammatory conditions contributes to platelet hyperreactivity and increased thrombotic risk [5].

Although accumulating experimental evidence supports the capacity of antioxidant compounds to modulate platelet reactivity, the precise molecular mechanisms linking antioxidant activity to receptor-level modulation and downstream signaling remain incompletely elucidated [5,6]. Whether plant-derived antioxidants exert their antiplatelet effects exclusively through indirect redox modulation or via direct molecular interactions with platelet receptors remains an open and mechanistically relevant question.

Plant-derived polyphenolic compounds constitute a structurally diverse class of bioactive molecules capable of engaging specific protein targets through defined molecular recognition events. Blueberries (*Vaccinium corymbosum* L.) represent a particularly rich phytochemical reservoir, characterized by high concentrations of anthocyanins and hydroxycinnamic acid derivatives, including cyanidin and delphinidin glycosides, as well as chlorogenic acid [7,8]. These compounds exhibit pronounced antioxidant and anti-inflammatory activities and have been inversely associated with cardiovascular disease incidence in both in vitro and epidemiological studies [8,9,10]. Importantly, emerging evidence indicates that polyphenolic bioactivity extends beyond free radical scavenging to include direct interactions with receptor and enzyme binding sites, emphasizing the need for mechanistic and structural-level investigations [4,11].

The biosynthesis of anthocyanins and hydroxycinnamic acid derivatives is strongly influenced by environmental stress conditions. Water deficit induces cellular oxidative stress in plants, triggering adaptive metabolic reprogramming that enhances phenylpropanoid pathway flux and secondary metabolite accumulation [12]. This response can be further potentiated by methyl jasmonate (MeJA), a key phytohormone that transcriptionally activates genes encoding enzymes involved in secondary metabolism [13]. Such agronomic strategies yield polyphenol-enriched blueberry extracts with increased chemical diversity, expanding the structural space of metabolites in terms of molecular mass, lipophilicity, conformational flexibility, and hydrogen bond donor–acceptor capacity [14,15].

Blueberries are particularly rich in structurally diverse phenolic compounds, including anthocyanins, flavan-3-ols, and hydroxycinnamic acids, which have been extensively associated with antioxidant and cardioprotective effects [7,8]. Among these, cyanidin-3-glucoside represents one of the predominant anthocyanins in the Vaccinium species, while catechin, chlorogenic acid, and caffeic acid are consistently reported as major flavanol and hydroxycinnamic acid derivatives contributing to total phenolic content. These compounds are not only quantitatively relevant but also biologically active, having demonstrated antioxidant, vasoprotective, and antiplatelet properties in both in vitro and in vivo models [6,16]. Notably, chlorogenic acid has been proposed to modulate platelet activation through cAMP-dependent pathways potentially linked to ADORA2A signaling. Based on their abundance, chemical representativeness, and documented cardiovascular relevance, these four phenolics were selected as molecular markers for quantitative profiling and subsequent in silico interaction analyses. From a structural and pharmacological perspective, ADORA2A represents an exemplary target for structure-based studies, as multiple high-resolution crystal structures capturing distinct functional conformational states are available [3,17]. These structures provide detailed insights into orthosteric binding site architectures, ligand-induced conformational rearrangements, and receptor activation mechanisms [3,4]. Importantly, the well-characterized structural plasticity of the ADORA2A orthosteric cavity suggests that, beyond synthetic xanthines and nucleoside analogs, small bioactive phytochemicals with appropriate hydrogen-bonding and aromatic features may also interact with this receptor [16]. In this context, accumulating pharmacological evidence has identified certain dietary phenolic acids as potential modulators of platelet signaling pathways. Among them, chlorogenic acid has been reported to suppress platelet activation through cAMP-dependent mechanisms compatible with ADORA2A-mediated signaling [16]. The involvement of this receptor has been further supported by studies employing selective ADORA2A antagonists and phosphodiesterase inhibitors [16]. However, whether chlorogenic acid represents the sole—or even the primary—polyphenolic mediator of ADORA2A-dependent antiplatelet effects remains unclear. Moreover, the extent to which water deficit and MeJA-induced metabolic reprogramming in blueberries alters the accumulation of additional phenolic compounds capable of interacting with ADORA2A has not been systematically explored. Addressing these gaps is critical for understanding how agronomically modulated phytochemical diversity translates into functional cardiovascular bioactivity. Although previous studies have shown that blueberry polyphenols possess antiplatelet activity and that methyl Jasmonate can modify secondary metabolism in plants, it remains unknown whether agronomic elicitation under contrasting water regimes alters selected bioactive phenolic compounds in a manner associated with platelet-modulating activity. Furthermore, it has not been investigated whether these agronomically influenced phenolic compounds possess structural features compatible with interaction with ADORA2A, a key receptor involved in platelet inhibition. Addressing this knowledge gap is essential for understanding how agronomic practices may influence the cardiovascular bioactivity of fruit-derived phytochemicals.

Based on this rationale, we hypothesized that water deficit and methyl Jasmonate modify the abundance of selected bioactive phenolic compounds in blueberry fruits and that these changes are associated with differences in platelet-modulating activity. We further hypothesized that representative phenolic compounds identified in these extracts possess structural compatibility with the human ADORA2A receptor, providing a plausible molecular basis for the observed biological responses. To test these hypotheses, we combined targeted HPLC-DAD analysis, antioxidant assays, platelet functional studies, and structure-based computational approaches. This integrative strategy provides a framework to investigate how agronomic practices influence selected bioactive phenolic compounds and their associated platelet-modulating activity, while generating structural hypotheses regarding potential receptor-level interactions.

## 2. Results

Neira et al. (2026) [18] reported that fruits of ‘Legacy’ exhibited the highest total phenolic content and ferric reducing antioxidant power (FRAP) among nine commercially relevant blueberry cultivars grown under field commercial conditions. Based on this evidence, ‘Legacy’ was selected as a model system to evaluate whether its antioxidant-related properties are maintained under controlled growing conditions and how they respond to environmental constraints associated with climate change. Additionally, exogenous application of methyl jasmonate (MeJA) has been shown to mitigate the negative effects of abiotic stress in blueberry plants, including water limitation, by modulating physiological and metabolic responses [15]. Building on these findings, the present study aimed to determine whether MeJA-mediated stress attenuation in potted, six-year-old blueberry plants translates into measurable changes in fruit antioxidant capacity, as a first step toward assessing potential health-related bioactivities.

### 2.1. Effect of MeJA Treatment and Water Availability on the Antioxidant Capacity of Blueberry Fruit Extracts

**Figure 1:**
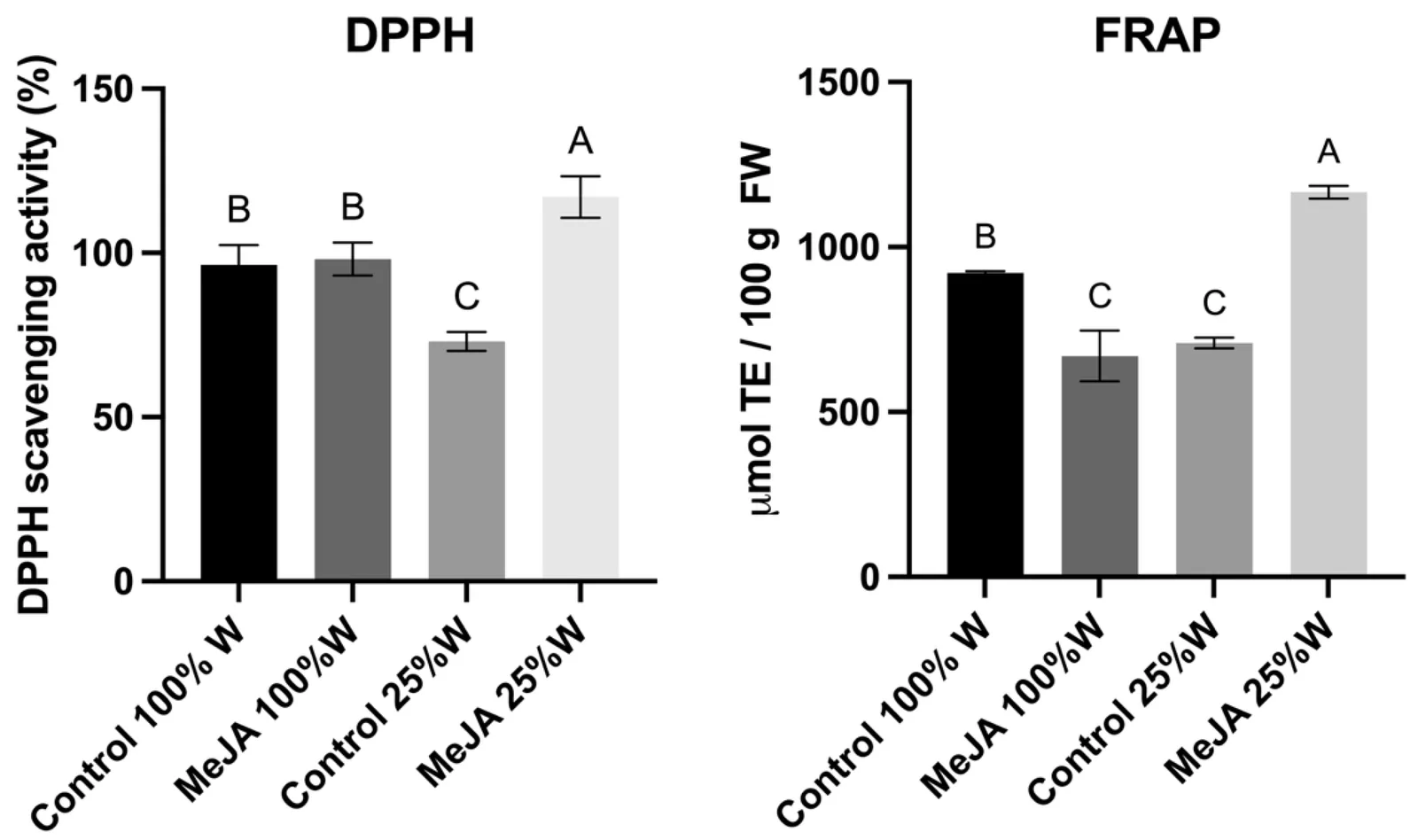
Effect of MeJA treatment on the activities of the antioxidant capacity in blueberry fruits. Different letters indicate significant differences (*p* < 0.05; one-way ANOVA). Bars represent means ± SE from three independent experiments.

The antioxidant capacity of blueberry fruit extracts obtained from control and MeJA-treated plants grown under contrasting water availability conditions was evaluated using DPPH scavenging and ferric reducing antioxidant power (FRAP) assays (Figure 1). Quercetin (100 µg mL^−1^), purchased from Merck (Sigma-Aldrich, St. Louis, MO, USA), was included as a positive control and exhibited 92% ±1.5% of DPPH scavenging activity, confirming the robustness of the antioxidant assays. Under well-watered conditions (100% water availability), DPPH radical scavenging activity did not differ significantly between control and MeJA-treated fruits (Figure 1). In contrast, FRAP values were significantly lower in MeJA-treated fruits than in the corresponding well-watered control (Figure 1).

Water restriction (25% water availability) affected both antioxidant assays. Compared with well-watered control fruits, water-deficit control fruits exhibited significantly lower DPPH scavenging activity and FRAP values (Figure 1). Under these conditions, MeJA application significantly increased both DPPH scavenging activity and FRAP values relative to untreated water-stressed plants, reaching the highest antioxidant values observed among all treatments (Figure 1). Overall, the antioxidant response differed according to the interaction between irrigation regime and MeJA treatment. Whereas MeJA had little effect under well-watered conditions, its application under water deficit was associated with increased antioxidant capacity as determined by both DPPH and FRAP assays.

### 2.2. Cytotoxic Activity on Platelets

**Figure 2:**
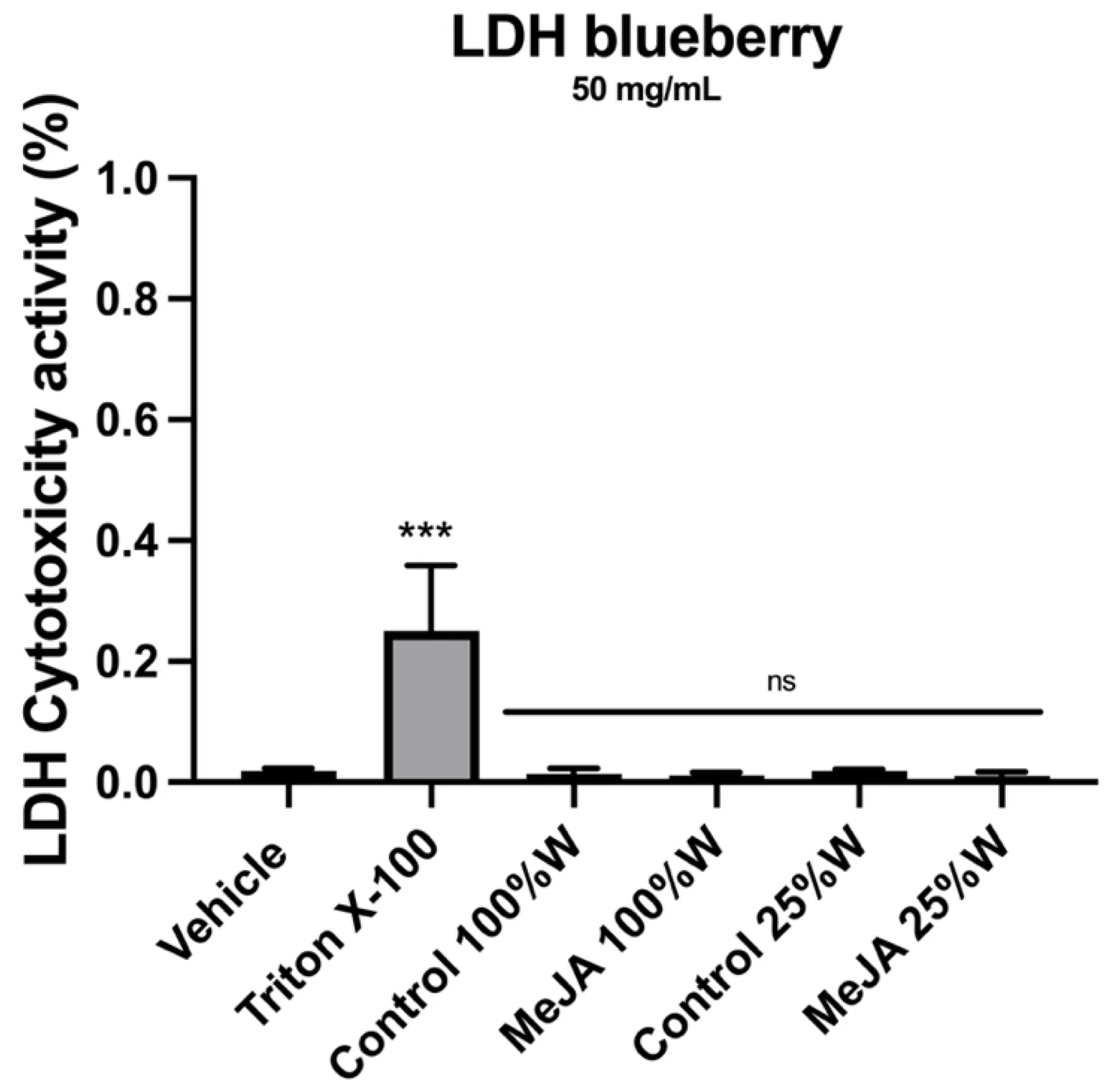
Cytotoxicity of blueberry extracts on human platelets. Washed platelets were incubated with blueberry extracts (50 mg/mL) for 10 min at 37 °C, and lactate dehydrogenase (LDH) release was measured to assess membrane integrity. Bars represent mean ± SE (*n* = 3). ns indicates no significant differences compared with the vehicle control (H_2_O). *** *p* < 0.001 versus Triton X-100 (positive control).

Before evaluating their antiplatelet activity, the potential cytotoxic effects of the blueberry extracts were assessed by measuring lactate dehydrogenase (LDH) release from washed human platelets (Figure 2). Platelets were incubated with the highest extract concentration tested (50 mg mL^−1^) for 10 min at 37 °C. As shown in Figure 2, none of the blueberry extracts induced a significant increase in LDH release compared with the vehicle control (H_2_O), indicating that platelet membrane integrity was preserved under all experimental conditions. No significant differences in cytotoxicity were observed among extracts obtained from different irrigation regimes or MeJA treatments.

Based on these results, all subsequent antiplatelet experiments were conducted using non-cytotoxic concentrations of the blueberry extracts.

### 2.3. Inhibition of Platelet Aggregation (IC_50_)

The inhibitory effect of blueberry extracts on platelet aggregation was evaluated in platelet-rich plasma stimulated with TRAP-6 (10 μM). Antiplatelet potency was quantified by determining the half-maximal inhibitory concentration (IC_50_) for each treatment (Table 1).

As shown in Table 1, extracts obtained from well-watered control plants (control 100%W) exhibited the greatest inhibitory potency, with an IC_50_ value of 7.5 mg mL^−1^. Water deficit alone moderately reduced this activity, increasing the IC_50_ to 9.7 mg mL^−1^ (control 25%W). In contrast, extracts obtained from MeJA-treated plants displayed higher IC_50_ values under both irrigation regimes (18.9 and 22.2 mg mL^−1^ for MeJA 100%W and MeJA 25%W, respectively), indicating lower inhibitory potency than their corresponding untreated controls.

**Figure 3:**
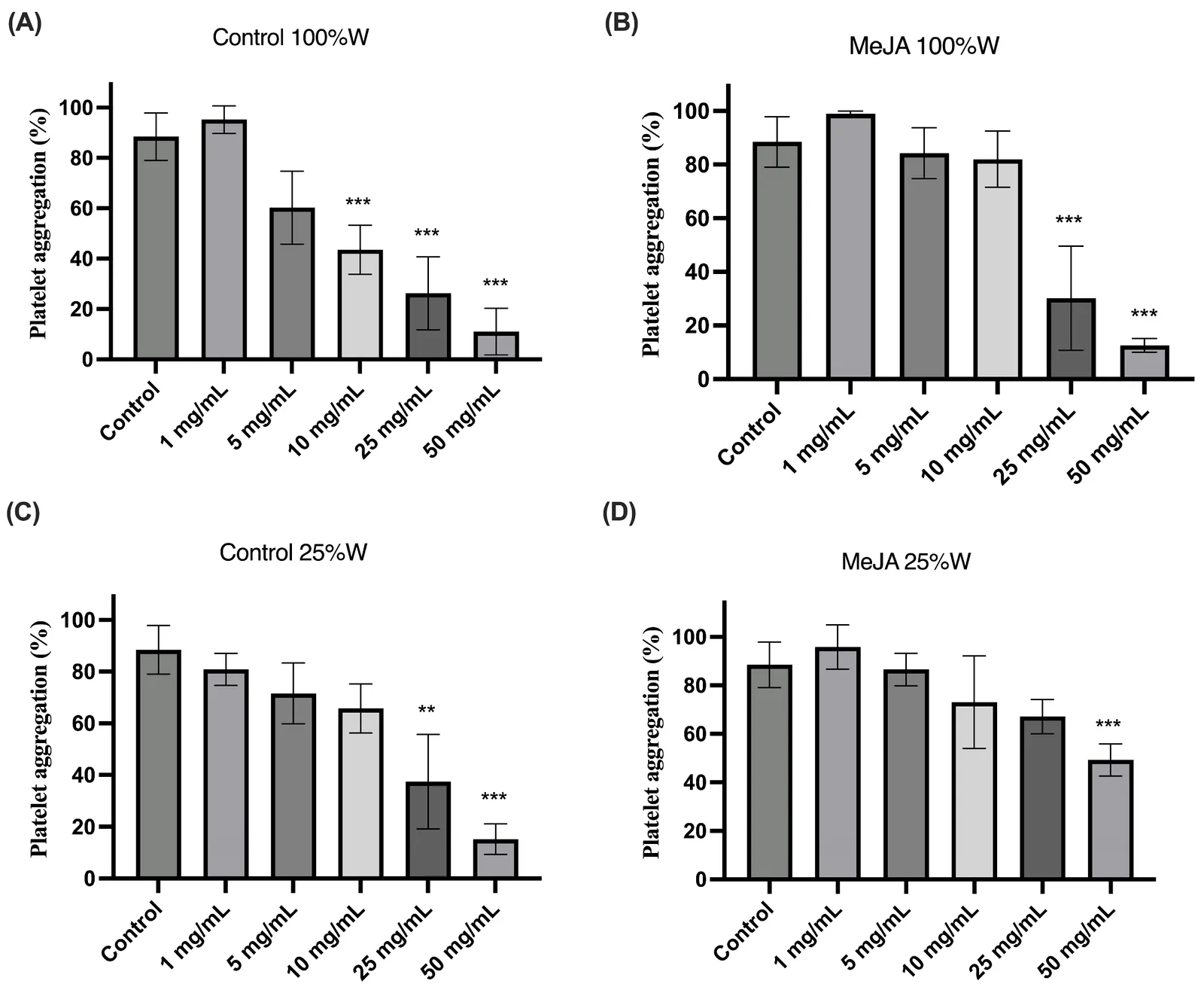
Modulation of platelet aggregation by blueberry extracts (E.Ard). Platelet aggregation was induced with TRAP-6 (10 µM) in the absence or presence of E.Ard. Platelet-rich plasma (PRP) was pre-incubated with the extracts at the indicated concentrations for 6 min at 37 °C prior to stimulation, and IC_50_ values were calculated for each extract. Bars show maximal aggregation expressed as a percentage of the activated control (mean ± SE, *n* = 6). “Basal” denotes unstimulated PRP (no TRAP-6). Statistical comparisons were performed by one-way ANOVA followed by a Bonferroni post hoc test. ** *p* < 0.01 and *** *p* < 0.001 indicate statistically significant differences versus the activated vehicle control (PRP + TRAP-6 + H_2_O).

Dose–response curves confirmed a concentration-dependent inhibition of platelet aggregation for all treatments (Figure 3). Under well-watered conditions, control extracts markedly reduced platelet aggregation from 10 mg mL^−1^ onward and produced near-complete inhibition at the highest concentration tested (50 mg mL^−1^). Extracts obtained from MeJA-treated plants under the same irrigation regime exhibited a similar concentration-dependent response, although higher concentrations were required to achieve comparable inhibition. Under water-deficit conditions, both control and MeJA extracts retained antiplatelet activity, but their inhibitory responses remained consistently lower than those observed for extracts from well-watered plants (Figure 3).

Overall, the platelet aggregation assays revealed distinct inhibitory potencies among treatments, with the strongest activity observed in extracts obtained from well-watered control plants, whereas MeJA-treated extracts consistently exhibited higher IC_50_ values irrespective of irrigation regime. These differences in antiplatelet activity prompted the subsequent characterization of selected phenolic compounds previously associated with cardiovascular bioactivity, with the aim of determining whether variations in their abundance were associated with the observed functional responses.

### 2.4. Effect of Blueberry Extracts on Platelet Activation

**Figure 4:**
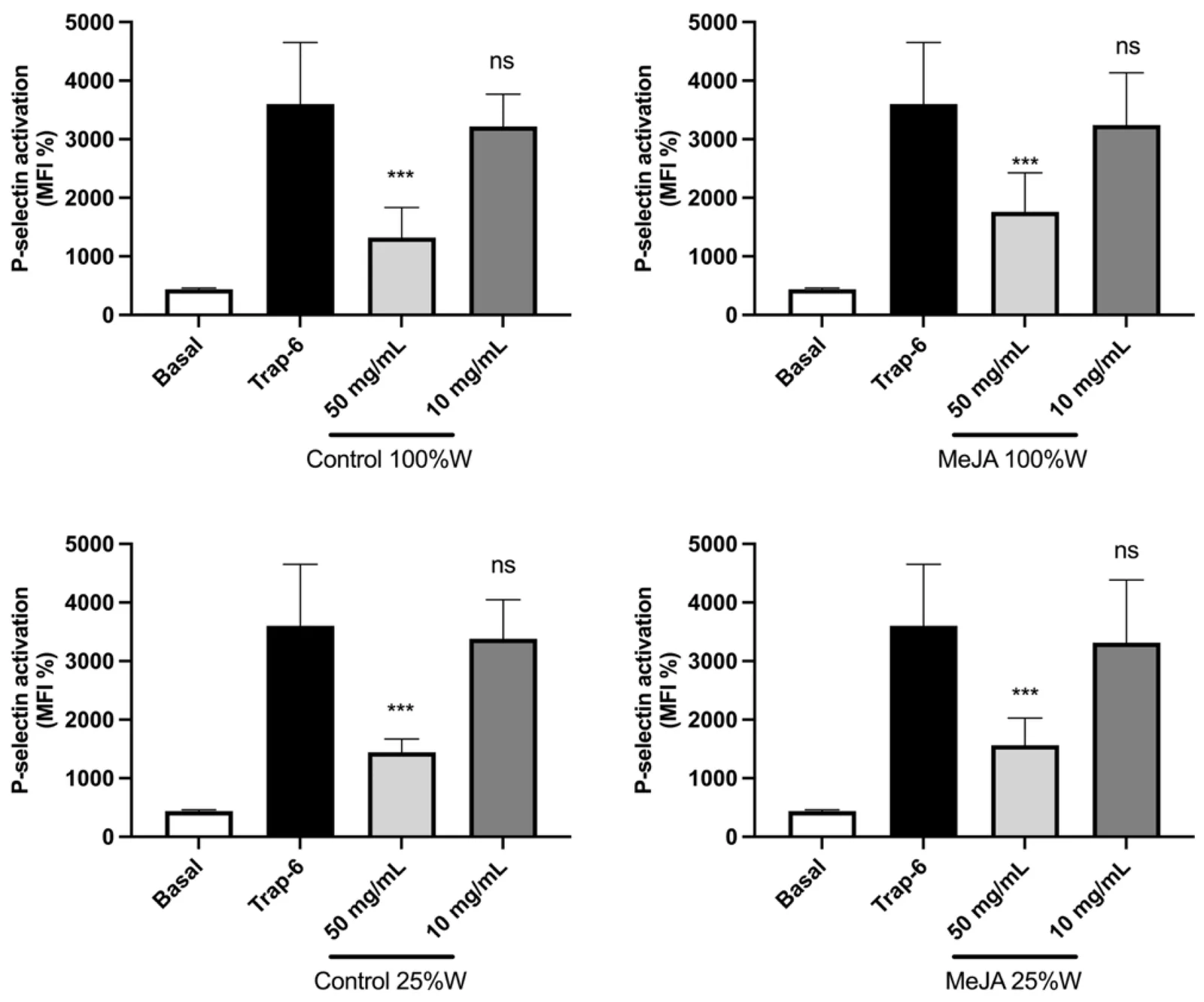
Effect of blueberry extracts on platelet activation assessed by P-selectin expression. Platelet-rich plasma was preincubated with blueberry extracts (10 and 50 mg/mL) for 10 min at 37 °C and then stimulated with TRAP-6 (10 µM). P-selectin (CD62P) surface expression was evaluated by flow cytometry using anti-CD61-FITC to gate the platelet population and anti-CD62P-PE as an activation marker. Bars represent mean ± SEM (*n* = 4). ns indicates no significant differences compared with the vehicle control (H_2_O). *** *p* < 0.001 vs. TRAP-6–stimulated control.

To further characterize the effect of blueberry extracts on platelet function, platelet activation was assessed by measuring the surface expression of P-selectin (CD62P) following stimulation with the PAR-1 agonist TRAP-6 (10 µM) (Figure 4). P-selectin is a well-established marker of α-granule secretion and platelet activation. As expected, stimulation with TRAP-6 markedly increased P-selectin expression compared with basal conditions (mean MFI = 3600.7 ± 428.6), confirming effective platelet activation. Preincubation with blueberry extracts reduced TRAP-6–induced P-selectin expression in a concentration-dependent manner (Figure 4). At the highest concentration tested (50 mg·mL^−1^), all extracts significantly decreased surface P-selectin expression relative to the stimulated control, producing approximately 50–60% inhibition irrespective of irrigation regime or MeJA treatment (Figure 4). In contrast, treatment with 10 mg·mL^−1^ resulted in only minor or non-significant reductions in P-selectin expression across all experimental groups.

Overall, all blueberry extracts retained the capacity to attenuate platelet activation at higher concentrations, whereas differences among agronomic treatments were less pronounced than those observed in the platelet aggregation assays. Given the differences observed in platelet aggregation but the comparatively similar effects on P-selectin expression, a targeted HPLC–DAD analysis was subsequently performed to quantify selected phenolic compounds previously associated with cardiovascular and antiplatelet activity.

### 2.5. Targeted HPLC-DAD Quantification of Selected Phenolic Compounds

**Figure 5:**
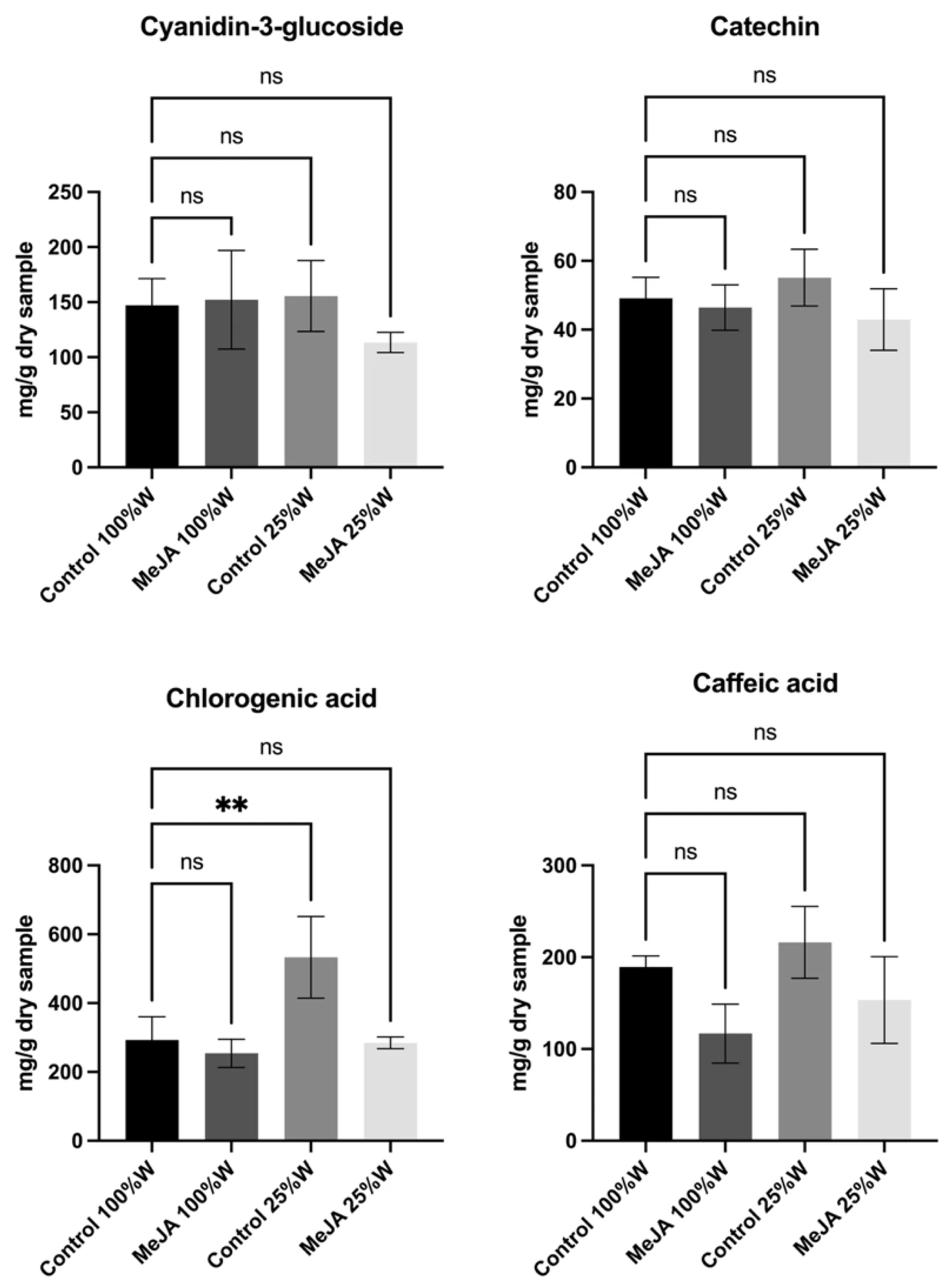
Targeted HPLC-DAD analysis of selected phenolic compounds in blueberry fruits grown under contrasting irrigation regimes with or without methyl Jasmonate (MeJA) treatment. Cyanidin-3-glucoside, catechin, chlorogenic acid, and caffeic acid were quantified using authentic reference standards. Data are presented as mean ± SD. Different letters indicate statistically significant differences among treatments (*p* < 0.05). The analysis highlights treatment-dependent differences in the abundance of selected phenolic compounds. ns indicates no significant differences compared with the control 100%W. ** *p* < 0.01 vs. control 100%W.

A targeted HPLC-DAD analysis was performed to quantify four phenolic compounds (cyanidin-3-glucoside, catechin, chlorogenic acid, and caffeic acid) selected based on their reported abundance in blueberry fruits and their previously described relevance to platelet-modulating and cardiovascular bioactivities [16]. The analysis revealed that the abundance of these compounds responded differentially to water deficit and MeJA treatment (Figure 5).

Cyanidin-3-glucoside concentrations did not differ significantly among irrigation regimes or MeJA treatments, although a trend toward lower levels was observed in MeJA-treated fruits under water-deficit conditions. Likewise, catechin content remained relatively constant across all treatments, with no statistically significant differences associated with either irrigation regime or MeJA application contrast, chlorogenic acid exhibited the most pronounced response among the compounds analyzed. Fruits obtained from water-deficit control plants (control 25%W) contained significantly higher chlorogenic acid levels than fruits from well-watered control plants (*p* < 0.01; Figure 5). This increase was not observed in MeJA-treated plants under water deficit, where chlorogenic acid concentrations remained comparable to those measured under well-watered conditions. Caffeic acid showed a similar tendency, with higher mean concentrations under water deficit in untreated plants, although these differences did not reach statistical significance.

Overall, the targeted HPLC-DAD analysis revealed compound-specific responses to irrigation regime and MeJA treatment rather than uniform changes across all quantified phenolics. These selected compounds were subsequently used for structure-based computational analyses because of their reported biological relevance and their differential abundance among treatments.

### 2.6. Structure-Based Analysis of Selected Phenolic-ADORA2A Interaction

To investigate the structural plausibility of receptor-level mechanisms underlying the observed platelet responses, the four phenolic compounds quantified by HPLC-DAD were further evaluated using structure-based computational approaches against the human adenosine A_2_A receptor (ADORA2A). Initial binding poses obtained by molecular docking were subjected to triplicate 100 ns molecular dynamics (MD) simulations. Structural stability, local conformational flexibility, and binding energetics were subsequently assessed using RMSD, RMSF, and MM-GBSA analyses, respectively. The co-crystallized antagonist ZM241385 was included as a reference ligand throughout all simulations.

#### 2.6.1. Structural Stability of Ligand–ADORA2A Complexes

**Figure 6:**
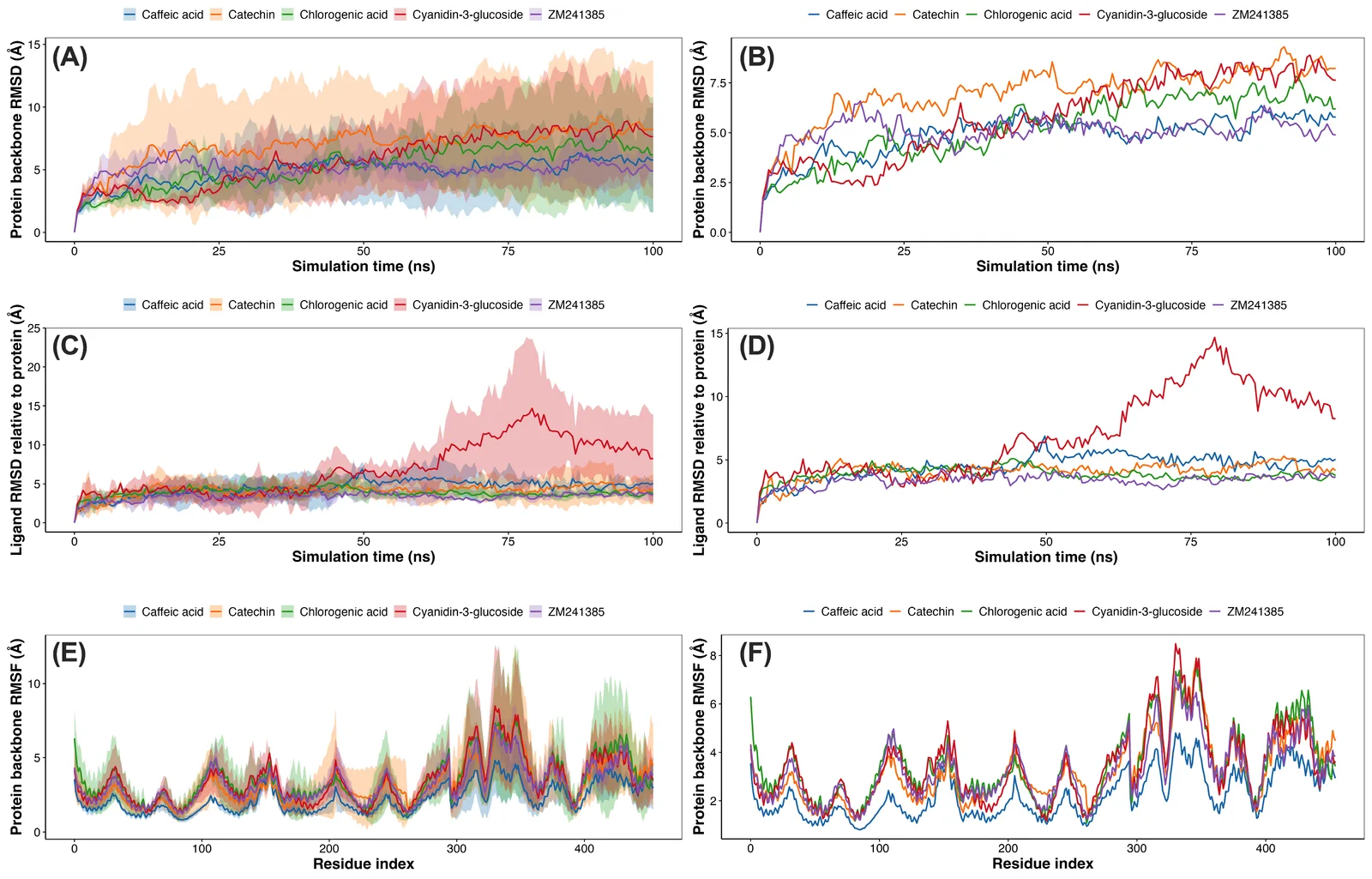
Molecular dynamics analysis of ligand–receptor complexes. (**A**,**C**) Mean root-mean-square deviation (RMSD) values ± standard deviation obtained from three independent simulations for selected phenolic ligands and the co-crystallized antagonist ZM241385. (**B**,**D**) Representative RMSD trajectories from individual simulations, illustrating the temporal stability of ligand positioning within the receptor binding pocket. RMSD values were calculated relative to the initial minimized structure after alignment of the protein backbone. (**E**,**F**) Root-mean-square fluctuation (RMSF) analysis of receptor residues over the simulation time, indicating local flexibility patterns in the presence of each ligand. Regions exhibiting comparable fluctuation profiles to the ZM241385-bound complex suggest preservation of overall receptor structural stability upon phenolic binding.

The structural stability of the ADORA2A–ligand complexes was evaluated through triplicate 100 ns MD simulations by monitoring the root mean square deviation (RMSD) of both the protein backbone and the bound ligands (Figure 6A–D). The co-crystallized antagonist ZM241385 exhibited stable RMSD values throughout the simulations, supporting the robustness of the simulation protocol and the structural integrity of the receptor model.

All selected phenolic ligands reached stable trajectories following an initial equilibration period and remained associated with the receptor binding pocket throughout the production phase, with no evidence of complete ligand dissociation (Figure 6). Mean RMSD trajectories and their corresponding standard deviations obtained from three independent simulations demonstrated good reproducibility across all systems.

Protein backbone RMSD analysis revealed ligand-dependent differences in receptor dynamics (Table S1). Among the evaluated systems, the ZM241385 complex displayed the lowest conformational variability (mean RMSD ≈ 5.0 Å), consistent with its experimentally resolved binding mode. Complexes containing caffeic acid and chlorogenic acid exhibited comparable backbone stability after equilibration, whereas catechin and cyanidin-3-glucoside were associated with larger RMSD fluctuations and broader conformational sampling during the simulations (Figure 6A,B).

Analysis of ligand RMSD further differentiated the stability of the individual binding modes (Figure 6C,D). Chlorogenic acid displayed the most stable behavior among the evaluated phenolics, maintaining RMSD values close to those of the reference antagonist throughout the simulations (Table S2). Catechin and caffeic acid exhibited intermediate mobility within the binding pocket, whereas cyanidin-3-glucoside showed the greatest positional variability, indicating increased conformational rearrangements during the simulation time.

Overall, the RMSD analyses indicate that all four phenolic compounds established persistent receptor-associated conformations during the simulations, although their dynamic behavior differed considerably. In particular, chlorogenic acid displayed the highest structural stability among the evaluated phenolics, whereas cyanidin-3-glucoside exhibited the greatest conformational mobility within the ADORA2A binding pocket.

#### 2.6.2. Ligand-Induced Local Flexibility of ADORA2A

To further investigate the effects of ligand binding on receptor dynamics, residue-level flexibility of ADORA2A was evaluated by calculating the root mean square fluctuation (RMSF) of protein backbone atoms over the MD trajectories (Figure 6E,F). RMSF profiles provide complementary information to RMSD analyses by identifying localized conformational fluctuations throughout the receptor structure.

Overall, all ligand–receptor complexes exhibited similar fluctuation profiles, with low mobility across most transmembrane regions and higher flexibility restricted to loop regions and protein termini, consistent with the intrinsic dynamic behavior of class A G protein-coupled receptors. This conserved pattern indicates that binding of the evaluated phenolic compounds did not induce major structural perturbations of the overall receptor architecture.

Quantitative analysis nevertheless revealed ligand-dependent differences in the magnitude of local flexibility (Table S3). Among the evaluated systems, the caffeic acid complex displayed the lowest average backbone fluctuations, indicating a comparatively rigid local conformation throughout the simulations. Intermediate RMSF values were observed for the ZM241385 and catechin complexes, both of which preserved receptor dynamics like those of the reference ligand. In contrast, chlorogenic acid and cyanidin-3-glucoside were associated with higher residue-level fluctuations, particularly within solvent-exposed loop regions, resulting in broader RMSF distributions (Figure 6E,F).

Importantly, although the magnitude of residue fluctuations varied among ligands, the positions of the principal RMSF peaks remained largely conserved across all systems. This observation suggests that ligand binding primarily modulated the amplitude of intrinsic receptor motions rather than generating new flexible regions or altering the overall structural organization of ADORA2A.

Collectively, the RMSF analyses indicate that the evaluated phenolic compounds differentially influenced local receptor dynamics while preserving the global structural framework of ADORA2A throughout the simulations.

#### 2.6.3. Binding Free Energy Analysis

**Figure 7:**
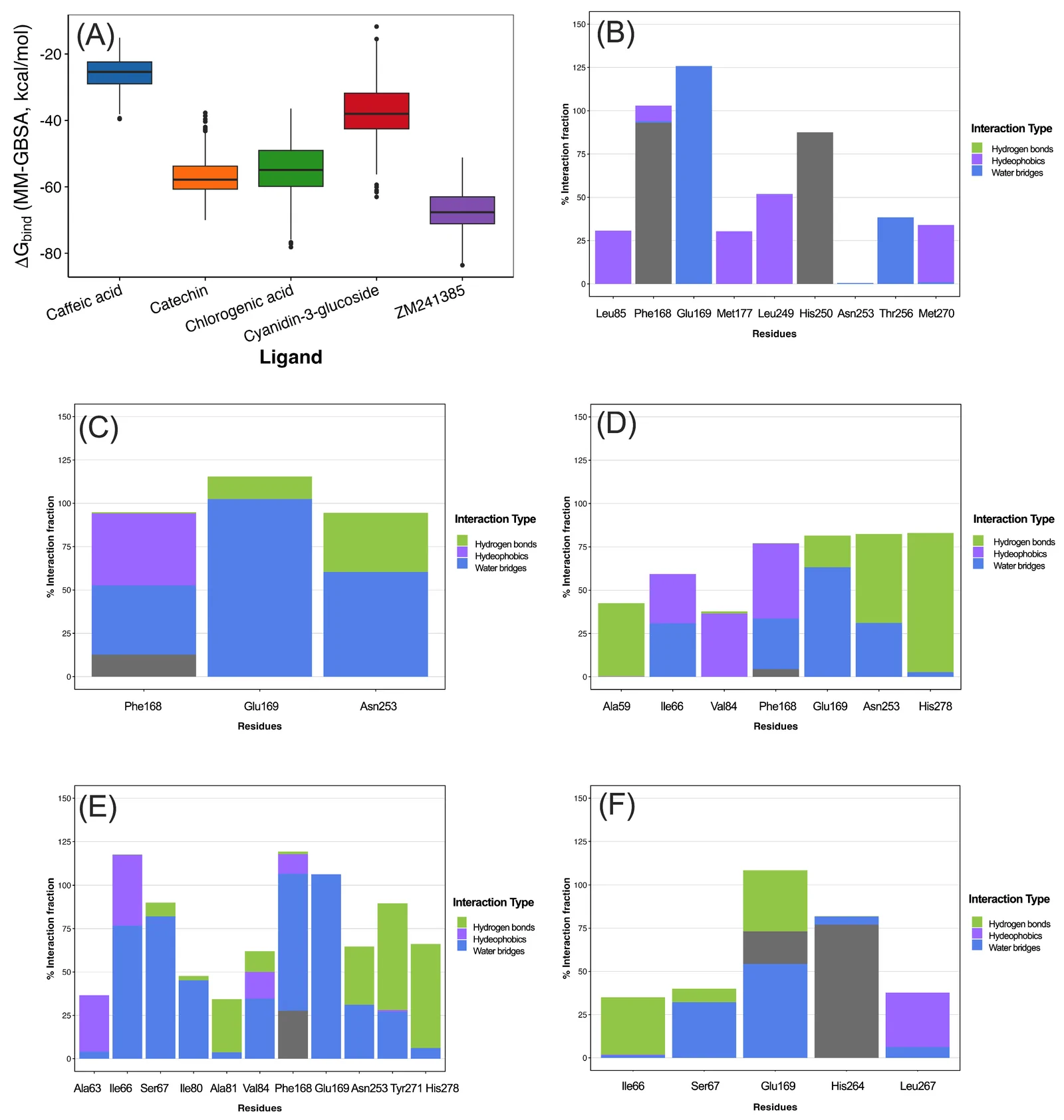
Binding free energy and residue interaction profile of phenolic ligands and the co-crystallized antagonist ZM241385 obtained from molecular dynamics simulations. (**A**) MM-GBSA binding free energy (ΔGbind) distribution calculated from the production phase of the trajectories for each ligand–receptor complex. Box plots represent the energetic stability of the complexes across simulation frames. (**B**–**F**) Per-residue interaction analysis derived from MD trajectories, showing the percentage of interaction fraction for hydrogen bonds, hydrophobic contacts, and water bridges between each ligand and key binding-site residues: (**B**) ZM241385, (**C**) catechin, (**D**) chlorogenic acid, (**E**) cyanidin-3-glucoside, and (**F**) caffeic acid. **In the interaction diagrams, gray indicates general van der Waals contacts or nonspecific hydrophobic interactions**. Interaction fractions represent the proportion of simulation time during which a given contact was maintained. Comparable interaction patterns between selected phenolics and ZM241385 indicate partial conservation of binding-site engagement and support their predicted affinity and structural stability within the receptor cavity.

To compare the energetic favorability of phenolic binding to ADORA2A, binding free energies (ΔG__bind_) were estimated using the Prime MM-GBSA approach on representative frames extracted from the MD trajectories (Figure 7A). As expected, the co-crystallized antagonist ZM241385 displayed the most favorable binding free energy, consistent with its experimentally resolved binding mode and its high structural stability throughout the simulations (Table S3). Among the evaluated phenolics, catechin and chlorogenic acid exhibited the most favorable binding energetics, with mean ΔG__bind_ values of −57.26 and −55.01 kcal·mol^−1^, respectively. Although both ligands displayed moderate energetic variability, their binding free energies were substantially more favorable than those of cyanidin-3-glucoside and caffeic acid, indicating comparatively stronger predicted interactions with ADORA2A. In contrast, cyanidin-3-glucoside showed less favorable binding energetics (mean ΔG__bind_ = −37.19 kcal·mol^−1^) together with the largest energetic dispersion among the evaluated ligands, suggesting greater variability in the sampled binding conformations. Caffeic acid presented the weakest predicted binding affinity (mean ΔG__bind_ = −25.97 kcal·mol^−1^), despite exhibiting a relatively narrow energy distribution throughout the simulations.

Overall, MM-GBSA calculations established a consistent energetic ranking of ligand binding, following the order: ZM241385 >> catechin ≈ chlorogenic acid > cyanidin-3-glucoside > caffeic acid (Table S3). This energetic trend was consistent with the structural stability observed during the MD simulations, particularly for chlorogenic acid and catechin, whereas cyanidin-3-glucoside and caffeic acid exhibited less favorable energetic profiles. Although MM-GBSA estimates do not provide experimental measurements of binding affinity, they support the structural plausibility of differential ligand stabilization within the ADORA2A binding pocket.

#### 2.6.4. Residue-Level Interaction Analysis

To further characterize the molecular basis of ligand recognition, protein–ligand interaction profiles were analyzed throughout the MD trajectories by quantifying hydrogen bonds, hydrophobic contacts, and water-mediated interactions involving residues within the ADORA2A orthosteric binding pocket (Figure 7B–F). Across all complexes, ligand stabilization involved a conserved set of residues previously implicated in ADORA2A ligand recognition, particularly Phe168, Glu169, and Asn253. However, the frequency and persistence of these interactions differed among the evaluated ligands, generating distinct interaction fingerprints. As expected, the reference antagonist ZM241385 exhibited the most persistent interaction network, characterized by stable water-mediated interactions with Glu169 together with recurrent hydrophobic contacts involving Phe168 and additional stabilization by Asn253 (Figure 7B). This interaction pattern is consistent with its experimentally resolved binding mode and the favorable energetic profile observed in the MM-GBSA analysis. Catechin and chlorogenic acid displayed more distributed interaction networks involving Glu169, Phe168, Ile66, Ser67, and Val84 (Figure 7C,D). In both complexes, water-mediated interactions represented a major contribution to ligand stabilization, although chlorogenic acid exhibited a more persistent interaction network than catechin, consistent with its lower ligand mobility observed during the MD simulations.

Cyanidin-3-glucoside established frequent contacts with Glu169 and Phe168 while also interacting with residues such as His278 and Tyr271 (Figure 7E). However, these interactions were more heterogeneous throughout the trajectories, reflecting the greater conformational flexibility of this ligand within the binding pocket.

Among the evaluated phenolics, caffeic acid displayed the simplest interaction pattern, with stabilization primarily supported by hydrogen bonds and water bridges involving Glu169 and Asn253 and relatively limited hydrophobic contacts (Figure 7F). This reduced interaction network is consistent with its comparatively less favorable MM-GBSA binding energy.

Taken together, the interaction analyses indicate that the evaluated phenolic compounds share several conserved recognition residues within the ADORA2A orthosteric pocket but differ in the persistence and organization of their interaction networks. These computational observations provide structural support for the predicted receptor binding modes while remaining consistent with the dynamic and energetic analyses presented above.

## 3. Discussion

### 3.1. MeJA-Induced Changes in Selected Phenolic Compounds and Phytochemical Quality

Jasmonates are recognized as central regulators of plant defense and secondary metabolism, particularly through activation of the phenylpropanoid pathway and downstream flavonoid biosynthesis [19]. Exogenous methyl jasmonate (MeJA) has been widely used as an elicitor to enhance phenolic accumulation in fruits and horticultural crops [20,21]. In blueberries, recent work by Neira et al. (2026) [18] demonstrated that the cultivar ‘Legacy’ exhibits superior total phenolic content and ferric reducing antioxidant power (FRAP) compared to multiple commercial cultivars. Our findings confirm that this cultivar maintains high antioxidant capacity under controlled pot conditions and further reveal that MeJA application under contrasting water regimes reshapes the qualitative phytochemical profile detected by HPLC. Interestingly, MeJA treatment did not uniformly enhance total antioxidant capacity as measured by FRAP and DPPH. Rather than a simple quantitative increase in total phenolics, our chromatographic analyses suggest a redistribution of specific phenolic subclasses. Similar qualitative shifts have been described in grapes, strawberries, and apples, where MeJA induced selective upregulation of flavonoids or hydroxycinnamic acids without necessarily increasing global phenolic indices [20,21,22,23]. This distinction is biologically relevant, as structural specificity—not total phenolic load—often determines bioactivity in mammalian systems. Because multiple phenolic compounds coexist within extracts, causal attribution to individual molecules cannot be inferred from compositional analysis alone. Thus, our data support the notion that MeJA acts as a metabolic modulator, potentially altering the relative abundance of bioactive compounds with differential cardiovascular relevance.

### 3.2. Antiplatelet Activity: Functional Relevance Beyond Antioxidant Capacity

Platelet activation plays a central role in arterial thrombosis and cardiovascular disease [24,25,26]. Dietary polyphenols have been reported to interfere with platelet aggregation, secretion, and receptor-mediated signaling [27,28]. Flavonoids such as quercetin and catechins modulate intracellular calcium signaling and inhibit granule secretion pathways. In this study, blueberry extracts significantly reduced TRAP-6–induced P-selectin (CD62P) surface expression in a concentration-dependent manner, without evidence of cytotoxicity. Because TRAP-6 specifically activates the protease-activated receptor-1 (PAR-1) pathway, the observed inhibition suggests interference with receptor-driven intracellular signaling cascades rather than nonspecific oxidative scavenging. Notably, control extracts exhibited lower IC_50_ values than MeJA-treated samples despite comparable antioxidant capacity. This dissociation between radical-scavenging power and antiplatelet potency reinforces the concept that cardiovascular bioactivity depends on specific structural classes rather than total antioxidant metrics. Similar observations have been reported in grape-derived polyphenols, where inhibition of platelet function correlated more strongly with phenolic acids and flavonols than with total polyphenol content [29].

The inhibition of TRAP-6–induced platelet activation observed in this study cannot be fully explained by nonspecific antioxidant activity, particularly given the lack of strict correlation with FRAP/DPPH values. Instead, the convergence between functional assays and computational binding stability supports a model in which selected phenolics interact with receptor-associated signaling axes, potentially influencing Gαs–adenylyl cyclase–cAMP pathways. Such redox–receptor interplay provides a mechanistic bridge between plant-derived metabolites and platelet signaling modulation. Our findings therefore suggest that agronomic modulation of secondary metabolism may alter the functional vascular effects of fruit-derived phytochemicals, even when classical antioxidant assays remain unchanged.

### 3.3. Structural Plausibility: Molecular Dynamics and Receptor Interaction

To explore a potential molecular basis for the observed platelet responses, we investigated the interaction of selected phenolic compounds with the crystal structure of the human ADORA2A receptor co-crystallized with the antagonist ZM241385 [30]. Molecular docking followed by molecular dynamics (MD) simulations was employed to evaluate ligand binding stability and interaction energetics under dynamic conditions that better approximate the physiological environment.

RMSD analyses across independent MD replicas indicated that several phenolic ligands maintained stable conformations within the receptor binding pocket, with deviations comparable to those observed for the co-crystallized antagonist. Likewise, MM-GBSA binding free energy estimations suggested favorable interaction energies, whereas residue decomposition analyses identified persistent contacts with amino acid residues involved in ligand stabilization.

Although these computational approaches cannot demonstrate receptor engagement or establish a biological mechanism on their own, they provide structural support for the hypothesis that selected blueberry phenolics can interact with the ADORA2A binding site. The consistency between the predicted binding stability and the concentration-dependent inhibition of platelet activation observed experimentally suggests that receptor-mediated modulation represents a plausible mechanism that warrants further experimental validation. Previous studies have shown that dietary flavonoids can interact with G protein-coupled receptors and modulate receptor-associated signaling pathways [31], supporting the biological relevance of investigating these interactions in blueberry-derived phenolics.

### 3.4. Integrative Perspective

Although receptor-level modulation cannot be experimentally confirmed within the scope of the present study, the computational analyses provide structural plausibility rather than direct evidence of receptor engagement that selected blueberry phenolics may interact with ADORA2A in a manner consistent with modulation of platelet signaling. These computational findings should therefore be interpreted as mechanistic support rather than direct experimental evidence.

Thus, the present work integrates plant physiology, targeted phytochemical characterization, platelet functional assays, and structure-based computational analyses to explore how agronomic practices may influence the biological properties of fruit-derived phytochemicals. Rather than demonstrating a direct causal relationship between changes in phenolic composition and platelet inhibition, our results indicate that agronomic treatments modify the abundance of selected bioactive phenolic compounds that are associated with altered platelet responses.

Functionally, blueberry extracts produced a concentration-dependent inhibition of TRAP-6-induced platelet activation, reflected by reduced P-selectin exposure without detectable cytotoxicity. These observations are consistent with previous reports showing that berry-derived phenolics—including anthocyanins, hydroxycinnamic acids, and flavan-3-ols—can attenuate platelet activation through multiple complementary mechanisms, including modulation of intracellular calcium signaling, cyclic nucleotide pathways, granule secretion, and integrin αIIbβ3 activation [28,29].

Collectively, these findings suggest that agronomic strategies designed to improve crop resilience may also influence the functional properties of fruit-derived phytochemicals. Future studies integrating untargeted metabolomics with quantitative bioactivity analyses will be valuable to identify the specific metabolites responsible for these effects and to better understand how agronomic interventions translate into cardiovascular bioactivity.

## 4. Materials and Methods

### 4.1. Plant Material, Extract Preparation and Phytochemical Characterization

#### 4.1.1. Plant Material

Six-year-old *Vaccinium corymbosum* L. ‘Legacy’ plants were cultivated in 30 L pots under controlled greenhouse conditions at INIA-Quilamapu (Chillán, Chile). The experiment followed a completely randomized design with six plants per treatment group. Irrigation was supplied via drip systems: control plants received 12 L every three days (100% water-holding capacity, WHC), while water-restricted plants received 3 L (25% WHC) on the same schedule. Soil moisture was monitored gravimetrically following the standardized method of Morales-Quintana et al. (2022) [32] to maintain consistent water regimes. Methyl jasmonate (MeJA) concentrations were selected based on previous studies [13,15,21], using 0.05% Tween-20. Foliar MeJA applications were performed at 10-day intervals from petal fall to fruit ripening (six applications in total) using an electric pump sprayer to ensure full leaf coverage. Control plants were sprayed with the same vehicle lacking MeJA. Random fruit samples (~100 berries per plant) were collected from upper, middle, and lower canopy zones, immediately frozen in liquid nitrogen, and stored at −80 °C until analysis.

#### 4.1.2. Blueberry Fruit Extract Preparation

The fruits were harvested in December 2024, frozen at −80 °C, and freeze-dried. After freeze-drying, the plant material was crushed in a mortar and stored at −20 °C. One gram of crushed fruit was weighed, and 10 mL of absolute ethanol (*v*/*v*) containing 1% citric acid (*v*/*v*) was added [33]. The mixture was subjected to ultrasound for 30 min (with a 10 min rest) and then passed through a 0.22 µm PTFE membrane filter. The filtrate was subjected to a new maceration process with the same volume of acidified ethanol over the next 24 h (this process was repeated for a total of 3 days). The supernatant was transferred into 15 mL tubes and centrifuged at 4000 rpm for 10 min at 4 °C. The aqueous phase was recovered again, and the sediment was discarded. The filtered acidified ethanol was then collected and concentrated in a rotary evaporator under reduced pressure.

#### 4.1.3. Total Antioxidant Capacity of the Blueberry Fruits

The antioxidant potential of fruit methanolic extracts was evaluated using two complementary assays based on radical scavenging and reducing capacity. Radical quenching activity was determined through the 1,1-diphenyl-2-picrylhydrazyl (DPPH) assay, adapted from Cheel et al. (2007) [34]. The antioxidant assay was validated using quercetin as a reference antioxidant. A quercetin calibration curve was generated under the same experimental conditions, and quercetin was included as a positive control in each experimental run to verify assay performance and provide a reference for radical scavenging activity. For this purpose, 20 µL of methanolic extract was added to a reaction mixture containing 0.25 mL of DPPH solution (0.5 mM) and 0.5 mL of acetate buffer (100 mM, pH 5.5). The reaction was allowed to proceed, and absorbance was recorded at 517 nm using a Multiskan SkyHigh microplate spectrophotometer (Thermo Fisher Scientific, Waltham, MA, USA). Antioxidant activity was expressed as the percentage of DPPH radical inhibition relative to a methanol-treated control. In parallel, the reducing capacity of the extracts was assessed using the ferric reducing antioxidant power (FRAP) assay, following the procedure described by Parra-Palma et al. (2020) [35]. In this assay, antioxidants present in the extracts reduce the ferric (Fe^3+^) form of the ferric–tripyridyltriazine (TPTZ) complex to its ferrous (Fe^2+^) form, generating a blue chromophore whose absorbance is proportional to the reducing power of the sample. Absorbance measurements were performed spectrophotometrically, and results were used as an indicator of total antioxidant capacity. For both assays, analyses were conducted in triplicate using pooled samples consisting of 15 ripe fruits per condition, with three independent biological replicates.

#### 4.1.4. Processing Samples for HPLC

Once dried, the extract was resuspended in 1 mL of a solution composed of 5% methanol with 1% formic acid (MeOH + FA 1%) and 95% water with 1% formic acid (H_2_O + FA 1%), corresponding to the initial mobile phase conditions used for HPLC analysis. The samples were subsequently filtered through a 0.22 µm membrane filter, and a 20 µL aliquot was injected into the HPLC system in all cases. Detection wavelengths were set at 280 and 520 nm for compound quantification, corresponding to the maximum absorption ranges of phenolic compounds and anthocyanins, respectively.

Chromatographic separation was carried out using a gradient elution method at 30 °C. The mobile phases consisted of solvent A (1% formic acid in water, H_2_O + FA 1%) and solvent B (methanol containing 1% formic acid, MeOH + FA 1%). The gradient program was as follows: 0–5 min, 5% B; 20 min, 30% B; 40 min, 50% B; 45–50 min, 97% B; 52 min, 5% B; and 57 min, 5% B. The flow rate was maintained at 1.2 mL min^−1^ throughout the analysis. Data acquisition and processing were performed using LabSolutions software (version 5.6, Shimadzu Corp., Kyoto, Japan). High-performance liquid chromatography coupled to a diode array detector (HPLC–DAD) analyses were performed using a Shimadzu Prominence system (Shimadzu Corporation^®^, Kyoto, Japan), equipped with an LC-20AT pump, an SPD-M20A diode array UV–Vis detector, and a CTO-20AC column oven. Separation was achieved using a reverse-phase C18 ODS-3 column (4.6 × 250 mm, 5 µm particle size; GL Sciences, Osaka, Japan). Calibration curves were constructed by plotting chromatographic peak area against analyte concentration and fitted by linear regression analysis. Analytical standards of cyanidin-3-glucoside chloride, catechin, caffeic acid, and chlorogenic acid were used for calibration and quantification. Cyanidin-3-glucoside chloride and chlorogenic acid were obtained from PhytoLab GmbH & Co. KG (Vestenbergsgreuth, Germany), catechin hydrate (≥98% purity, HPLC grade) was purchased from Sigma-Aldrich (St. Louis, MI, USA), and caffeic acid (≥98.0% purity, HPLC grade; product code C0625) was also obtained from the same supplier. Individual stock solutions were prepared by accurately weighing each standard and dissolving it in the initial mobile phase, followed by sonication to ensure complete dissolution. Working solutions were prepared by serial dilution of the stock solutions. All calibration levels and samples were injected in quadruplicate (*n* = 4), and the mean peak area was used for quantification. The resulting calibration equations were as follows: cyanidin-3-glucoside, y = 16,870x − 104,325 (R^2^ = 0.9996); catechin, y = 76,956x − 911,966 (R^2^ = 0.9194); caffeic acid, y = 1930.5x + 130,579 (R^2^ = 0.9805); and chlorogenic acid, y = 13,349x + 116,897 (R^2^ = 0.9927). All graphs and statistical analyses were performed using GraphPadPrism v10.4.1.

### 4.2. Biological Activity and Platelet-Related Functional Assays

#### 4.2.1. Preparation of Human Platelets

Blood samples were obtained from healthy donors who had abstained from anti-inflammatory medication for at least seven days. Informed consent was obtained from all donors, and the study was approved by the Ethics Committee of the University of Talca (Folio 06-2021). Blood was collected in citrate tubes and centrifuged at room temperature for 12 min at 1200 rpm to obtain platelet-rich plasma (PRP). The PRP was then centrifuged at 3000 rpm for 10 min at room temperature to yield platelet-poor plasma (PPP). Platelet counts were determined using a Mindray BC-3000 Plus Hematology Counter (Shenzhen Mindray Bio-Medical Electronics Co., Ltd., Shenzhen, China) [36].

#### 4.2.2. Cytotoxic Activity

Washed platelets were prepared to assess cytotoxicity. Blood was collected using acid-citrate-dextrose (ACD, 4:1 *v*/*v*) and centrifuged at room temperature for 12 min at 1200 rpm to obtain PRP. The PRP was centrifuged at 3000 rpm for 10 min, and the resulting platelet pellet was resuspended in calcium-free Tyrode’s buffer containing ACD (5:1 *v*/*v*). The platelets were washed by centrifugation at 3000 rpm for 10 min. Washed platelets (approximately 3 × 10^8^ platelets/mL) were incubated with blueberry extracts (E.Ard) at 50 mg/mL for 10 min at 37 °C. After incubation, the platelets were centrifuged at 3000 rpm for 8 min, and the supernatant was analyzed for lactate dehydrogenase (LDH) release using a cytotoxicity assay kit (Cayman Chemical, Madison, WI, USA). Following centrifugation, 50 µL of the resulting supernatant was subjected to the LDH assay, and absorbance was measured at 490 nm. A 0.3% Triton X-100 solution was used as a positive control [37].

#### 4.2.3. Inhibition of Platelet Aggregation

The inhibition of platelet aggregation was evaluated using a microplate-based assay with slight modifications. PRP was incubated with blueberry extracts (E.Ard) at various concentrations (1, 5, 10, 25, and 50 mg/mL) or with a vehicle control (water) at 37 °C for 6 min. Platelet aggregation was then induced by adding thrombin, a receptor-activating peptide (TRAP-6, 10 µM). The samples were transferred to 96-well plates (Greiner, Stonehouse, UK) and shaken at 1200 rpm for 5 min at 37 °C using a plate shaker (Thermo Scientific, MA, USA). PRP plus vehicle (water) served as the control for maximum aggregation. Absorbance was measured at 450 nm using a spectrophotometer (Agilent BioTek, Winooski, VT, USA). The percentage of platelet aggregation was calculated as follows:

- Platelet aggregation (%) = 100 × [(Control Absorbance (PRP) − Absorbance with E.Ard)/(Control Absorbance (PRP) − Blank Absorbance (PPP))].

The percentage inhibition of platelet aggregation was then determined by:

- Inhibition (%) = 100 − [(Aggregation (%) of E.Ard/Aggregation (%) of negative control) × 100] [38].

#### 4.2.4. Inhibition of Platelet Activation

Platelet activation was assessed by preincubating PRP for 10 min at 37 °C with blueberry extracts at 50 and 10 mg/mL or with vehicle (water). Platelet aggregation was then induced by adding TRAP-6 (10 µM). Platelet activation was evaluated by incubating 30 µL of each sample for 30 min at room temperature in the dark with saturating concentrations of anti-CD61-FITC (a platelet marker) and anti-CD62P-PE (P-selectin expression). The anti-CD61-FITC antibody allowed precise gating of the platelet population. Samples were subsequently analyzed by flow cytometry using a BD FACSLyric cytometer (BD Biosciences, Nueva Jersey, CA, USA). Platelet populations were gated based on forward scatter (FSC) and side scatter (SSC) along with CD61 positivity to distinguish them from background noise, as previously described in the literature [39].

### 4.3. In Silico Analysis of Phytochemical–Platelet Receptor Interactions

#### 4.3.1. Protein Structure Selection and Preparation

The human ADORA2A receptor was selected as the molecular target for docking studies. A comprehensive search was conducted in the Protein Data Bank [40], identifying 98 experimentally resolved ADORA2A structures from *Homo sapiens*. These structures were initially ranked according to structural quality metrics, including resolution and completeness. The top 25 structures were subsequently subjected to detailed visual inspection to evaluate the presence of engineered mutations, missing residues, or structural artifacts.

Among these candidates, only one structure was identified as fully mutation-free: PDB ID 3EML [30], determined by X-ray crystallography at a resolution of 2.6 Å. This structure is co-crystallized with the selective A_2_A receptor antagonist ZM241385, bound within the orthosteric ligand-binding pocket, making it particularly suitable for docking validation and comparative studies. Protein preparation was performed using the Protein Preparation Wizard implemented in the Schrödinger Maestro suite [41]. Missing hydrogen atoms were added, bond orders were assigned, and steric clashes were resolved through restrained energy minimization. Protonation states of ionizable residues were assigned using PROPKA [42,43] at physiological pH (7.0), ensuring a chemically consistent representation of the receptor under near-physiological conditions. Final optimization of the protein structures was carried out using the OPLS3e force field [44].

#### 4.3.2. Ligand Selection and Preparation

Four naturally occurring polyphenolic compounds: cyanidin-3-glucoside, catechin, chlorogenic acid, and caffeic acid, were downloaded from ChEMBL [45,46] and selected as candidate ligands for docking. The co-crystallized antagonist ZM241385 was included as a reference control to validate the docking protocol and provide a benchmark for comparative energetic analysis. Ligand structures were prepared using the LigPrep module of Maestro. All ligands were geometrically optimized under the OPLS3e force field, and their ionization and tautomeric states were generated using Epik at pH 7.0 ± 0.5. For each compound, the most probable protonation state at physiological pH was retained for subsequent docking calculations.

#### 4.3.3. Grid Generation and Docking Protocol

The receptor grid was generated based on the experimentally defined binding site observed in the 3EML crystal structure. Key residues involved in stabilizing the co-crystallized ligand, Phe168, Glu169, and Asn253, were used to define the center of the docking grid. The outer grid box was set to 16 Å, while the inner grid box was restricted to 10 Å, ensuring adequate coverage of the orthosteric binding pocket while maintaining spatial specificity. Molecular docking was performed using the Glide module in Extra Precision (XP) mode to enhance discrimination between closely related binding poses and reduce false positives. Default XP docking parameters were employed, allowing full ligand flexibility while keeping the receptor rigid. For each ligand, multiple binding poses were generated, and the top-ranked pose based on the Glide docking score [47,48] was selected for further analysis.

The Glide XP docking score, which integrates terms accounting for van der Waals interactions, electrostatics, hydrogen bonding, hydrophobic contacts, and desolvation effects, was used as the primary energetic criterion for comparative evaluation of ligand binding affinity relative to the reference antagonist ZM241385.

#### 4.3.4. Molecular Dynamics Simulations

All-atom molecular dynamics (MD) simulations were performed to evaluate the structural stability and dynamic behavior of the ADORA2A–ligand complexes obtained from molecular docking. Five systems were considered: the four selected polyphenolic ligands (cyanidin-3-glucoside, catechin, chlorogenic acid, and caffeic acid) and the reference antagonist ZM241385. In all cases, simulations were conducted using the crystallographic structure of ADORA2A (PDB ID: 3EML), starting from the most energetically favorable docking pose identified for each ligand. MD simulations were carried out using the Desmond MD engine implemented in the Schrödinger Maestro suite. The OPLS3e force field was employed consistently for the protein, ligands, and ions. Each protein–ligand complex was solvated in an explicit SPC water model using an orthorhombic simulation box, ensuring a minimum distance of 10 Å between any protein atom and the box boundaries. Systems were electrically neutralized by the addition of Na^+^ and Cl^−^ counterions; no additional salt concentration was imposed. System preparation followed the standard Desmond relaxation protocol, which includes an initial energy minimization and short equilibration stages under restrained conditions to remove unfavorable contacts and allow solvent relaxation. No positional restraints were applied during the production phase.

Production MD simulations were performed in the NPT ensemble for 100 ns per replica, with three independent replicas generated for each system to ensure statistical robustness. Temperature was maintained at 300 K using a Langevin thermostat, while pressure was controlled at 1 atm using the Martyna–Tobias–Klein barostat (Regents of the University of California, University of California San Francisco, San Francisco, CA, USA). A time step of 2 fs was employed, with constraints applied to covalent bonds involving hydrogen atoms.

Post-simulation analyses were conducted using tools provided within the Schrödinger suite. Protein structural stability was assessed by calculating the root-mean-square deviation (RMSD) of the protein backbone relative to the initial equilibrated structure. Ligand stability within the binding pocket was evaluated through the ligand RMSD relative to the protein. Residue-level flexibility was characterized by computing the root-mean-square fluctuation (RMSF) of protein backbone atoms.

Binding free energy estimates were obtained using the Prime MM-GBSA approach applied to representative frames extracted from the MD trajectories. In addition, protein–ligand interaction profiles were analyzed along the trajectories to characterize the persistence and nature of key intermolecular interactions, including hydrogen bonds and hydrophobic contacts.

All analyses were performed independently for each replica, and results are reported as mean values with associated standard deviations where applicable.

## 5. Conclusions

Exogenous methyl jasmonate modulated the abundance of selected bioactive phenolic compounds in *Vaccinium corymbosum* ‘Legacy’ grown under contrasting water regimes without proportionally altering the overall antioxidant capacity of the extracts. Despite comparable FRAP and DPPH values, blueberry extracts exhibited concentration-dependent inhibition of TRAP-6-induced platelet activation, indicating that platelet-modulating activity was not directly associated with global antioxidant capacity.

Targeted HPLC-DAD analysis, combined with platelet functional assays and molecular dynamics simulations, identified representative phenolic compounds that may contribute to the observed biological activity and supported the structural plausibility of their interaction with the ADORA2A receptor. Together, these findings provide evidence that agronomic practices can modify selected bioactive phenolics associated with the vascular bioactivity of blueberry extracts, while highlighting the need for future metabolomic and quantitative correlation studies to identify the individual metabolites responsible for these effects.

## Figures and Tables

**Table 1:** Estimated IC_50_ (mg·mL^−1^) for each extract (*n* = 5 donors). Lower IC_50_ indicates greater potency.

Blueberry Extracts	IC_50_ (mg/mL)
Control 100%W	7.5
MeJA 100%W	18.9
Control 25%W	9.7
MeJA 25%W	22.2

## Data Availability

The original contributions presented in this study are included in the article. Further inquiries can be directed to the corresponding authors.

## Supplementary Materials

The following supporting information can be downloaded at https://www.mdpi.com/article/10.3390/ijms27167306/s1.
