# Supplementary material for: Water Deficit and Methyl Jasmonate Enhance the Antiplatelet Potential of Blueberries Through Changes in Selected Phenolic Compounds
Source: Int J Mol Sci. 2026 Aug 16;27(16):7306. doi: 10.3390/ijms27167306 (PMC13513635; doi:10.3390/ijms27167306)
Supplement: Supplementary file 1 [file ijms-27-07306-s001.zip › ijms-4468965-supplementary.pdf]

Table S1: Root-mean-square deviation (RMSD) of protein during MD simulation.

Ligand	n	Min	Q1	Median	Mean	Q3	Max	SD
ZM241385	606	0	4.28	5.08	5.06	5.76	9.13	1.243
Caffeic acid	606	0	2.94	4.02	4.87	6.68	10.63	2.50
Chlorogenic acid	606	0	3.50	3.50	5.28	6.05	13.98	2.88
Cyanidin-3-glucoside	606	0	2.99	4.14	5.77	8.28	13.52	3.50
catechin	606	0	3.29	5.99	7.00	11.18	14.71	3.99

Table S2: Root-mean-square deviation (RMSD) of ligand during MD simulation.

Ligand	n	Min	Q1	Median	Mean	Q3	Max	SD
ZM241385	606	0	2.87	3.35	3.37	3.89	5.96	0.80
Caffeic acid	606	0	3.33	3.79	3.86	4.28	7.49	0.88
Chlorogenic acid	606	0	3.01	3.88	4.11	5.17	8.39	1.50
Cyanidin-3-glucoside	606	0	3.29	4.40	4.48	5.71	8.90	1.56
catechin	606	0	3.88	6.14	6.96	8.18	25.36	4.48

Table S3: XXXX (MM-GBSA) binding Free Energy.

Ligand	n	Min	Q1	Median	Mean	Q3	Max	SD
ZM241385	606	-83.62	-71.13	-67.67	-67.35	-63.01	-51.18	5.75
Caffeic acid	606	-70.04	-60-70	-57.85	-57.85	-53.76	-37.73	5.40
Chlorogenic acid	606	-78.17	-59.87	-54.93	-55.01	-49.05	-36.42	7.72
Cyanidin-3-glucoside	606	-63.04	-42.53	-38.02	-37.19	-31.79	-11.75	8.16
catechin	606	-39.59	-28.99	-25.40	-25.97	-22.40	-15.09	4.76
